# Survival Benefit of Primary Tumor Treatment in Uveal Melanoma: A Re-Analysis of the Collaborative Ocular Melanoma Study (COMS) and Natural History Study (NHS) Cohorts

**DOI:** 10.3390/cancers16223839

**Published:** 2024-11-15

**Authors:** Hans Witzenhausen, Gustav Stalhammar

**Affiliations:** 1 St. Erik Ophthalmic Pathology Laboratory, St. Erik Eye Hospital, 17164 Stockholm, Sweden; 2 Department of Clinical Neuroscience, Division of Eye and Vision, Karolinska Institutet, 17177 Stockholm, Sweden; 3 Ocular Oncology Service, St. Erik Eye Hospital, 17164 Stockholm, Sweden

**Keywords:** uveal melanoma, choroidal melanoma, prognosis, survival, metastasis, deferral, primary tumor treatment

## Abstract

Objective: To evaluate whether primary tumor treatment provides a survival benefit in uveal melanoma by comparing patients who declined treatment (Natural History Study, NHS) with those who received treatment in the Collaborative Ocular Melanoma Study (COMS) for medium-sized choroidal melanomas. Methods: Individual-level survival data were reverse-engineered from cumulative all-cause mortality curves in the original COMS and NHS publications. Censoring patterns were estimated from numbers at risk and descriptive statistics. A Bonferroni-corrected significance level of 0.017 was applied. Additionally, to ensure a conservative approach, NHS cohort data were iteratively adjusted by reducing the 8-year cumulative mortality by one percentage point if the Cox regression hazard ratio for all-cause mortality, the unadjusted risk ratio for death, and the 95% confidence intervals (CIs) of the Kaplan–Meier curves did not show a smaller survival difference than originally reported. Results: Kaplan–Meier analysis revealed significantly higher cumulative mortality in the NHS cohort compared to the COMS cohort (log–rank *p* = 0.012). When restricting the analysis to the first 8 years to account for unclear censoring patterns beyond this period, the NHS cohort still demonstrated worse survival (*p* = 0.008). A sensitivity analysis, varying censoring times by ±25% over 1000 iterations, confirmed worse survival in the NHS cohort in 100% of cases. Conclusions: In this re-evaluation, patients who declined treatment for medium-sized choroidal melanomas had significantly worse survival, suggesting a potential survival benefit of primary tumor treatment.

## Simple Summary

Uveal melanoma is a life-threatening cancer of the eye. There has been ongoing debate about whether treating the main tumor in the eye can actually help patients live longer. In our study, we compared previously published results from patients who received treatment for their eye tumor with those who chose not to receive treatment. By analyzing survival data from both groups, we discovered that patients who did not undergo treatment had a higher risk of death. This finding suggests that treating the primary eye tumor can provide a significant survival benefit for patients with uveal melanoma. Our results also emphasize the importance of timely treatment and may influence future medical guidelines and decisions, ultimately improving patient care for this serious eye cancer.

## 1. Introduction

In 2003, Straatsma and colleagues evaluated the survival outcomes for 42 patients eligible for the Collaborative Ocular Melanoma Study randomized trial for medium-sized choroidal melanomas (COMS) who declined enrollment and chose not to undergo treatment [1,2]. Patients who initially deferred treatment but later received melanoma therapy within 45 days of COMS eligibility were excluded from the analysis, which was conducted as part of the Natural History Study (NHS). Of the 42 patients, 22 ultimately opted for treatment (eye-conserving radiation therapy or enucleation) at a mean of 1.4 years after declining initial participation (standard deviation (SD) 1.4).

The five-year all-cause mortality was 30% (95% confidence interval (CI), 18–47%) for NHS patients, compared with 18% (95% CI, 16–20%) for COMS patients. The unadjusted risk ratio for death in NHS patients was 1.79 (95% CI, 1.08–2.97), which decreased to 1.54 (95% CI, 0.93–2.56) after adjusting for baseline age and tumor size. While these results suggested a higher mortality rate in untreated patients, the authors emphasized that the findings were probative but not conclusive evidence of a survival benefit from treatment. The clinical importance of demonstrating a survival benefit from primary tumor treatment, or the lack thereof, cannot be overstated, as it would define the intervention as either life-saving or purely palliative.

The statistical methods employed consisted of Kaplan–Meier survival estimates and Cox proportional hazards modeling. However, Cox regression generates hazard ratios, not risk ratios. Hazard ratios compare the rate of events (e.g., death) between groups over time, while risk ratios compare the overall proportion of events over a fixed period. It is possible that Straatsma and colleagues mistakenly referred to hazard ratios as risk ratios. This distinction is important, as hazard ratios are more appropriate for time-to-event analyses. Notably, no risk ratios were provided for later time points beyond 5 years, even though additional mortality events occurred, seemingly at a higher rate in the NHS cohort. Additionally, while Kaplan–Meier survival curves were generated, no test statistics or *p*-values were reported to assess the significance of the survival differences between the NHS and COMS cohorts.

Subsequent literature has debated the survival benefits of timely treatment for primary tumors and in some cases, the benefit of treatment at all [3,4,5,6,7,8]. To address these concerns, we re-evaluated the NHS and COMS cohorts and performed a statistical comparison of their survival curves to assess the survival implications of deferring treatment in medium-sized choroidal melanoma.

## 2. Materials and Methods

Patients in the COMS and NHS had been evaluated at 43 clinical centers located in the United States and Canada [9]. Individuals with a clinically confirmed diagnosis of choroidal melanoma by a COMS-certified ophthalmologist between November 1986 and 31 July 1998 were reported to the COMS Coordinating Center in Baltimore, Maryland. The diagnosis was based on a comprehensive ophthalmic examination, which included best-corrected visual acuity, tumor size and location assessment, A-scan and B-scan ultrasonography, fundus photography, and fluorescein angiography. To be eligible for COMS (which also included a trial of pre-enucleation radiation versus no radiation for patients with large melanomas undergoing enucleation, an observational study of small tumors, reports of radiation-induced conditions, second primary cancers, etc., but herein only refers to the trial of enucleation versus iodine-125 plaque brachytherapy for patients with medium-sized tumors), patients had to have a choroidal melanoma measuring 2.5 to 10.0 mm in apical height and no more than 16.0 mm in basal diameter. Between November 1986 and November 1990, the inclusion criteria allowed for tumors between 3.1 and 8.0 mm in apical height. Patients with peripapillary tumors (within 2.0 mm from the optic disk) were included only if plaque placement could cover the entire tumor and an appropriate margin. In addition, patients had to meet several general eligibility criteria: a primary choroidal melanoma in one eye, be 21 years of age or older, have no other primary tumor, and have no serious concurrent illness expected to limit survival to less than five years. The best-corrected visual acuity in the fellow eye had to be 20/200 or better. Patients with previous treatment for choroidal or ciliary body melanoma, or any condition secondary to the tumor, were excluded, as were those with significant extra scleral extension (>2.0 mm), as described in the original publications [10,11].

The NHS cohort included patients who were eligible for the COMS trial but declined treatment or enrollment. Patients who initially declined but subsequently received melanoma treatment within 45 days of COMS eligibility were excluded from the NHS. For patients who died, medical reports, histopathological data, and laboratory findings were reviewed by the COMS Mortality Coding Committee to determine metastatic status [1].

To obtain raw data, we contacted all named authors of the original publication by email in 2021. The second author responded but no longer had access to the dataset. Attempts to contact the other authors were unsuccessful, and in 2024, the data were reverse-engineered as follows:

Figure 2 from the original publication (figure titled “Kaplan–Meier curves of the cumulative proportion of patients with medium choroidal melanoma who died by time after determination of eligibility for the COMS medium melanoma trial; enrolled COMS medium tumor trial patients NHS patients with 95% CI”, reprinted with permission from Elsevier [1]) was uploaded to the Plot Digitizer Online App (version 3.1.5, https://plotdigitizer.com/app, accessed on 6 September 2024). This tool allows for data extraction from graphs. A minimum of 60 data points were obtained from each cumulative proportion curve for the NHS and COMS cohorts. Two datasets were generated, each with x-values (years after enrollment/decline of enrollment) and y-values (cumulative percent dead from any cause), creating an initial dataset representing 1317 COMS patients and 42 NHS patients, with mortality proportions identical to the original figure. The data collection process is illustrated in Supplementary Figure S1.

**Figure 1:**
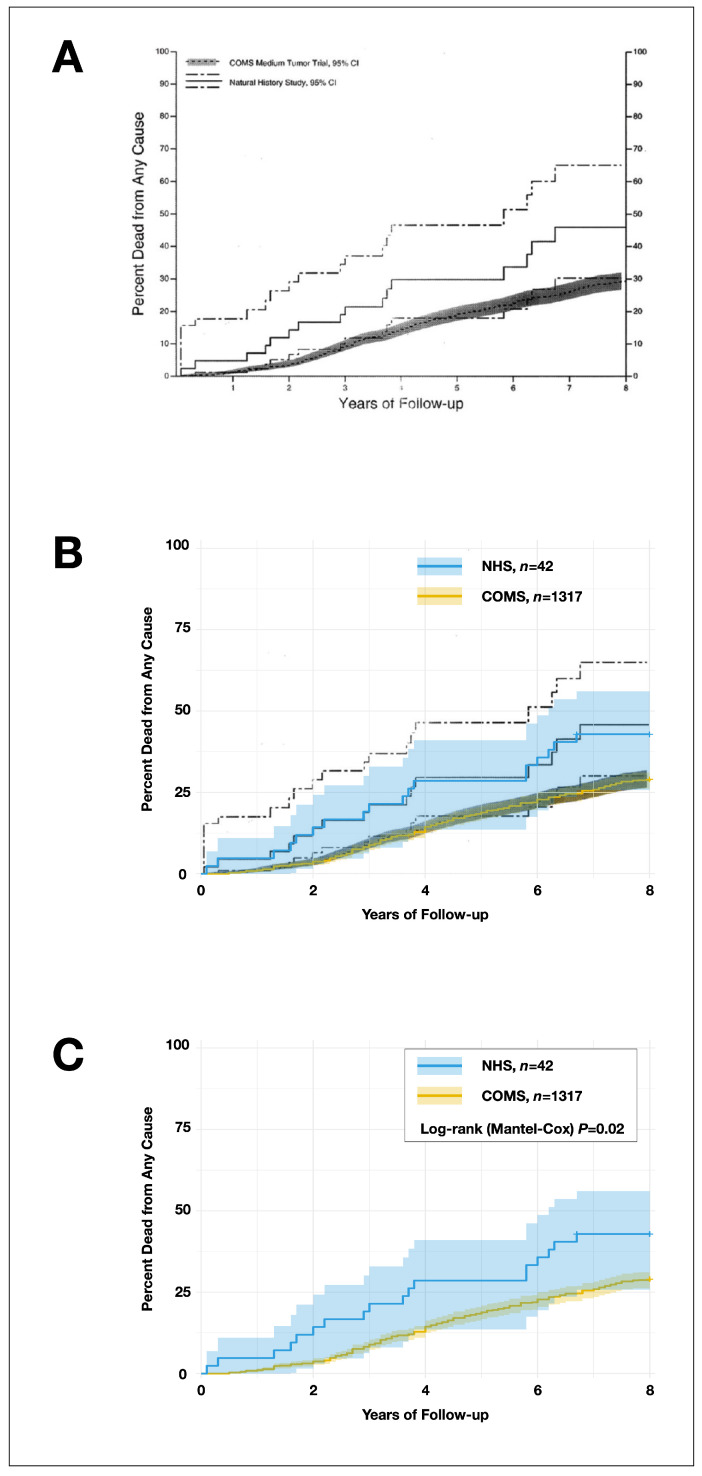
Kaplan–Meier survival curves show the cumulative proportion of deaths from any cause in the NHS and COMS cohorts. (**A**) The original figure from the 2003 publication, which did not include formal statistical comparisons of the curves. Dashed lines represent limits of the 95% confidence intervals (Cis) (**B**) Overlaying the reverse-engineered survival curves for the NHS and COMS cohorts onto the original curves revealed that the 95% CIs for the reverse-engineered data were closer than in the original study, confirming our conservative methodology. (**C**) Despite this, the log–rank (Mantel–Cox) test yielded a *p* of 0.02. Note that these analyses do not account for censoring, which will be addressed in the next step (Figure 2). The figure from the original publication was reprinted with permission from Elsevier [1]. COMS, Collaborative Ocular Melanoma Study. NHS, Natural History Study.

Censoring rates were estimated using the number-at-risk table from the 2006 COMS publication, which reported the 12-year survival for patients treated with plaque brachytherapy or enucleation (Figure 1A, titled “Cumulative percentage of patients dead [from all causes] by time since enrollment and treatment arm”) [12]. The reduction in the number at risk, minus deaths, was considered the number of censored events per interval and assigned to the midpoint of each interval [13]. As explicit censoring data for the NHS cohort were unavailable, estimates were derived using published survival figures, narrative data on median follow-up and range, and the number of patients with known vital status at 5 and 10 years from the original study, as detailed in the “Censoring and Sensitivity Analysis” section. The final individual-level dataset is provided in Supplementary Table S1.

This study adhered to the tenets of the Declaration of Helsinki. As declared in the Swedish Ethical Review Act (2003:460), approval from the Swedish Ethical Review Authority was not required as this study involved the re-analysis of publicly available data [14]. It did not involve any treatments, tests, examinations, interviews, or other interventions with study subjects. Additionally, it did not affect the subjects physically or psychologically, did not involve biological samples, and did not include any data or sensitive information that could be traced back to any individual.

### Statistical Methods

Kaplan–Meier survival curves were generated for the NHS and COMS cohorts using the survival, survminer, and readxl packages in R (R Foundation for Statistical Computing, version 4.4.1, Vienna, Austria). Survival distributions between the cohorts were compared using the log–rank (Mantel–Cox) test. To ensure accuracy, the log–rank test results were cross-verified using SPSS Statistics, version 29.0 (Armonk, NY, USA). To control for Type I errors, the significance threshold was adjusted using the Bonferroni correction, dividing the conventional 0.05 level by 3 (reflecting the number of statistical tests yielding *p* values). Thus, a two-sided *p*-value of less than 0.017 was considered statistically significant. As an additional measure of conservativeness, we required that the lower limit of the 95% confidence interval (CI) for the Kaplan–Meier curve we generated for the NHS cohort be lower than the corresponding lower limit of the 95% CI reported in the original publication at all time points [1]. This adjustment ensured that we were assessing a smaller survival difference between the NHS and COMS cohorts than was reported in the original comparison. To verify this, we calculated 8-year risk ratios and Cox proportional hazards ratios between the NHS and COMS cohorts. If these ratios for the NHS cohort were not lower than those reported in the original publication, or if the 95% CIs of the Kaplan–Meier curves did not indicate a smaller survival difference, we iteratively adjusted the NHS cohort data by reducing the 8-year cumulative mortality by one percentage point until these criteria were met.

## 3. Results

### 3.1. Descriptive Statistics

As reported in the original study, the 1317 patients enrolled in the COMS trial had a mean age of 60 years at the time of enrollment, while the 42 patients in the NHS cohort had a mean age of 66 years. Data on the anatomical extent of the primary tumor, such as ciliary body involvement or extraocular extension, were not provided. However, patients with predominantly ciliary body melanomas or extraocular extension greater than 2 mm were excluded from both cohorts as dictated by the study design. At baseline, COMS tumors were larger than NHS tumors, with a mean largest basal diameter (LBD) of 11.4 mm versus 10.3 mm and a mean thickness of 4.8 mm versus 3.8 mm, respectively.

### 3.2. Risk Ratios

The initial Cox proportional hazards model produced a hazard ratio of 1.51 (95% CI, 0.92–2.50) for NHS patients, with an 8-year risk ratio of 1.31 (95% CI, 0.69–1.93). Both these estimates were lower than those reported in the original publication, consistent with our pre-specified approach that required smaller survival differences than those in the original study.

### 3.3. Initial Kaplan–Meier Curves

The Kaplan–Meier survival curve of the cumulative proportion of deaths in the NHS and COMS cohorts, as reported in the original publication [1], was compared to the Kaplan–Meier curves generated from our reverse-engineered datasets. The comparison confirmed that the separation between our newly generated curves and their 95% confidence intervals was smaller than in the original publication, supporting our more conservative approach.

Although the curves were less separated, the log–rank (Mantel–Cox) yielded a log–rank *p* of 0.02, Figure 1. These results did not account for censoring, which will be addressed in the next step, and are not fully reliable. However, as the primary effect of censoring is on the variability and precision of the Kaplan–Meier curve—particularly the 95% confidence intervals—and given that these intervals in our analysis closely resembled those from the original publication, the impact of properly accounting for censoring may be limited.

### 3.4. Censoring and Sensitivity Analysis

**Figure 2:**
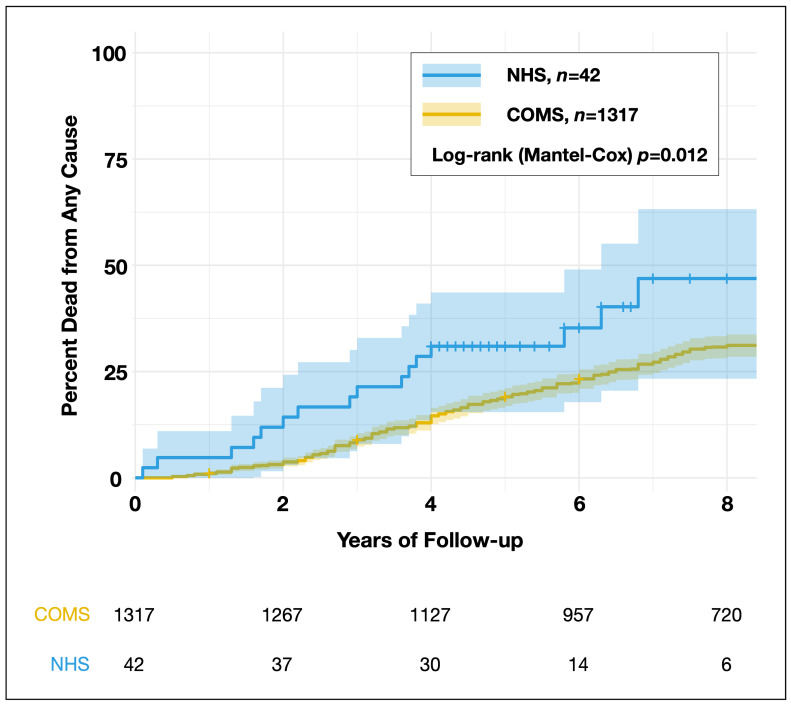
Kaplan–Meier survival curve showing the cumulative proportion of deaths from any cause in the NHS and COMS cohorts, accounting for censoring assumptions. The number at risk for the COMS cohort was extracted from the original publication’ s reported data. For the NHS cohort, censoring rates were estimated from the published survival figure and narrative data, including median follow-up, range, and the number of patients with known vital status at 5 and 10 years. COMS, Collaborative Ocular Melanoma Study. NHS, Natural History Study.

In the final step, we performed a Kaplan–Meier survival analysis incorporating censoring rates for the COMS cohort based on the available number-at-risk table from the original study (e.g., 1317 patients at 0 years, 1301 at 1 year, 1267 at 2 years, etc.). Since explicit censoring data for the NHS cohort was not available, we estimated censoring rates using information derived from the published survival curves, narrative data on median follow-up time, range, and the number of patients with known vital status at 5 and 10 years, as reported in the original study. Specifically, the study stated: “Based on time since COMS eligibility, vital status was known at 5 and 10 years for 32 and 20 of the 42 NHS medium melanoma patients, respectively, […] a total of 16 medium melanoma patients had died”. From this, we inferred that among the original 42 NHS patients, 16 had died, 4 were still alive at 10 years, and 22 had unknown vital status, indicating that they were censored earlier than 10 years post-COMS eligibility. At 5 years, 10 patients had unknown vital status. The median follow-up was 5.3 years (range 4–10.7 years), suggesting that 10 patients were censored between years 4 and 5, and the remaining 12 were censored between 5 and 10.7 years. Therefore, in our reverse-engineered cohort, the 22 patients with unknown vital status at 10 years were censored between 4 and 9 years, the 23rd patient was censored at 10.7 years (to achieve an identical median follow-up of 5.3 years), and the remaining 2 patients were censored at 11 years. The 720 COMS patients who remained at risk at 8 years were also censored at 11 years. The reconstructed individual-level data are provided in Supplementary Table S1. A Kaplan–Meier analysis of this dataset yielded a log–rank test *p*-value of 0.012 (Table 1, Figure 2). Even when accounting for the unclear censoring pattern beyond 8 years through a life-table analysis restricted to the 0–8 year period, the NHS cohort demonstrated significantly worse survival than the COMS cohort (Wilcoxon [Gehan] *p* = 0.008), reinforcing the robustness of this finding.

To further test the robustness of the survival difference to variations in censoring assumptions, we performed a sensitivity analysis. In this analysis, censoring times for both cohorts were randomly varied by ±25% over 1000 iterations. For each iteration, we generated a Kaplan–Meier survival curve and performed a log–rank test to assess whether the NHS cohort had worse survival than the COMS cohort. The sensitivity analysis revealed that in 100% of the iterations, the NHS cohort exhibited worse survival outcomes compared to the COMS cohort. This consistency across all iterations highlights that the observed survival difference is robust to variations in censoring assumptions, confirming that NHS patients consistently experienced poorer survival than COMS patients, irrespective of the censoring strategy applied.

## 4. Discussion

In this study, we re-evaluated the results from the landmark Collaborative Ocular Melanoma Study, particularly focusing on the Natural History Study cohort, comprising patients who declined treatment. Despite several factors that could have reduced the chances of observing a statistically significant survival benefit of treatment—such as smaller baseline tumor sizes in NHS patients and our adjustments to minimize survival differences beyond those reported in the original publication to reduce the risk of type I error—we found that survival outcomes remained significantly worse for the NHS cohort. This finding reinforces the hypothesis that primary tumor treatment provides a survival benefit in uveal melanoma.

These findings have critical implications for the clinical management of primary uveal melanoma. If treatment is believed to reduce the risk of metastasis and subsequent death, it not only underscores the importance of primary tumor treatment—potentially shifting the perception from ocular treatment being purely palliative to it having a curative role for some patients—but also highlights the significance of early detection and treatment [15]. If the prognosis worsens the longer a uveal melanoma remains untreated, timely intervention becomes essential, given the high propensity of uveal melanoma to metastasize, typically with fatal consequences [16,17]. On the other hand, if one argues against a survival benefit from primary tumor treatment, the urgency of early detection diminishes, and the potential adverse effects of prompt treatment, such as visual impairment, would weigh more heavily in treatment decisions [3].

Assuming that treatment does confer a survival benefit, healthcare systems must enhance screening protocols for early detection of small melanomas and refine strategies to distinguish between low- and high-risk lesions. Decentralized surveillance programs, utilizing optometrists or opticians with advanced imaging technologies such as fundus photography and optical coherence tomography (OCT), supported by remote consultations with experts and deep-learning algorithms, could play a pivotal role in expanding early detection efforts [18,19,20]. Furthermore, genetic and molecular profiling through biopsy techniques and chromosome 3 testing of circulating tumor cells (CTC) could help identify lesions with higher metastatic potential, providing critical insights for more personalized treatment and surveillance strategies [21,22,23].

It is evident that treatment cannot be initiated for tumors that have not yet been detected, and some observation for growth is necessary to differentiate between benign and malignant choroidal melanocytic lesions. However, once melanoma is diagnosed, delaying treatment may allow additional tumor cells to disseminate from the eye. Continued growth of the primary tumor also provides the opportunity to acquire further aggressive traits, potentially accelerating the growth rate of any metastases seeded after such events. By treating the primary tumor promptly, we may prevent additional tumor cell spread and mitigate further worsening of prognosis.

Some studies have not observed a significant relationship between treatment delays and prognosis, including Damato et al., who concluded that “Deferring treatment of choroidal melanomas until documentation of growth may delay iatrogenic visual loss by months or years and is associated with minimal increase in metastatic mortality, at least with small tumors with usual growth rates of up to 40% per year” [6,24]. However, this interpretation now warrants reevaluation: The 4.3% yearly growth rate observed for 24 tumors by Damato et al. would correspond to a volume doubling time of approximately 2008 days, assuming a semi-spheroidal shape and similar growth in thickness and diameter, which is in exceptionally slow, as demonstrated in our meta-analysis [8]. In fact, even the reported worst-case scenario involving an annual growth rate of 40%, corresponding to a doubling time of 752 days, is still rather slow. This rate aligns with a metastatic death risk increase of 1.3% per month in 40-year-old females with a 10-mm diameter monosomy-3 melanoma. In other words, this analysis, performed by an independent research team with independent datasets, draws similar conclusions about the prognostic impact of treatment delays, when taking the differences in assumed growth rate into account.

Furthermore, some previous observations may have been slightly overinterpreted in the field. For example, reports of *BAP1* mutations and other canonical aberrations arising in an early, punctuated burst do not necessarily preclude the possibility of later aberrations that could further accelerate metastasis growth [25]. Similarly, findings of shared mutations between primary tumors and metastases should not be misread as indicating that all metastases ultimately cause patient death are invariably seeded very early. Nor should calculations based on constant doubling time assumptions imply that metastases resulting in patient death always “occur at a time when the primary uveal melanoma is too small to be clinically detected” [26,27].

Shain and colleagues observed that while “metastatic dissemination occurred early during the development of the primary tumor”, they also identified additional driver mutations arising in later stages, which were shared with metastases [28]. These later mutations included *CDKN2A*, loss of chromosome 3 (including *PBRM1*), gain of 8q, and gain of 1p. Supplementary data from Shain’s study suggest that 5 of 35 (14%) patients had metastases originating from later branches in the phylogenetic trees (cases A11, A16, A50, A59, and A60). Two of these patients (A50 and A60) had small, potentially asymptomatic tumors, making earlier detection less likely.

This suggests two important points: first, that if effective primary tumor treatment, such as enucleation, had been performed in the three remaining cases with larger tumors before these additional genetic aberrations developed, the specific metastases that were analyzed could have been prevented (though not necessarily others); and second, that if tumor cells had already been seeded from older parts of these three tumors and managed to establish growing macrometastases, these cells may have carried fewer driver mutations, potentially resulting in slower growth.

### Limitations

This study has several limitations. First, our results rely on reverse-engineered survival data reconstructed to approximate the original COMS study outcomes. Despite considerable efforts to ensure the validity of the simulated NHS cohort, discrepancies between real-world data and the reverse-engineered data may have introduced bias. Although the timing of mortality events could be deduced from the survival plots, the reverse-engineering approach could not precisely capture the timing of censoring events in the NHS cohort. Instead, these were estimated based on the available descriptive statistics, including median follow-up, range, and the number of patients with known vital status at 5 and 10 years. This means that while the exact timing of censoring events is not fully accounted for, the estimated censoring should be reasonably accurate.

Additionally, comparing survival between two reverse-engineered cohorts cannot account for potentially unbalanced risk factors between the COMS and NHS cohorts, as data on additional risk factors were not available and thus could not be reverse-engineered. This represents a significant limitation, similar to the Kaplan–Meier curves in the original publication, which also did not account for risk factors.

Future studies could address the limitations of our approach by conducting prospective studies that collect real-world survival data directly from patients who decline treatment. Such studies would enable a more accurate assessment of survival outcomes without the potential biases of data reconstruction. Additionally, expanding access to large, multi-institutional databases may allow for more robust comparisons between treated and untreated cohorts, incorporating key genetic and molecular markers.

Lastly, the original COMS study itself had inherent limitations, including potential selection bias. Patients who declined treatment may have differed from those who enrolled, particularly in unmeasured factors such as chromosome 3 or 8q aberrations, BAP1 mutations, and gene expression profiles—key prognostic markers in uveal melanoma. Notably, NHS patients were older and presented with smaller tumors at baseline, which are positive and negative prognostic factors, respectively [16,29,30]. 

## 5. Conclusions

In this re-evaluation, patients who declined treatment for medium-sized primary choroidal melanomas had significantly worse survival compared to those treated promptly, suggesting a potential survival benefit of primary tumor treatment.

Clinically, these findings indicate that treating uveal melanoma at an earlier stage may reduce the risk of metastasis and improve overall survival. The results also suggest that healthcare systems should prioritize early detection, focusing on identifying the few malignant choroidal melanocytic lesions among the many benign counterparts to enable prompt treatment.

However, factors such as patient age and tumor size may influence these findings, as the original publication found that statistical significance was negated when adjusted for in regression analyses. Future studies should address the limitations of reverse-engineered data by focusing on prospective, real-world data collection. Ultimately, definitive evidence of causality would require a randomized clinical trial, though such a study would present ethical challenges.

## Figures and Tables

**Table 1:** Kaplan–Meier Overall Survival (OS) Point Estimates with 95% Confidence Intervals for COMS and NHS Cohorts.

COMS					NHS			
Time (Years)	Number at Risk	Kaplan–Meier OS Estimate	95% CI Lower	95% CI Upper	Number at Risk	Kaplan–Meier OS Estimate	95% CI Lower	95% CI Upper
1	1306	99%	98%	99%	40	95%	89%	100%
2	1267	96%	95%	97%	37	86%	76%	97%
3	1200	91%	90%	93%	34	79%	67%	92%
4	1127	85%	84%	87%	30	69%	56%	85%
5	1054	81%	79%	83%	20	69%	56%	85%
6	957	77%	74%	79%	14	65%	51%	82%
7	763	73%	70%	75%	8	53%	37%	77%
8	720	69%	66%	72%	6	53%	37%	77%

CI, Confidence Interval. COMS, Collaborative Ocular Melanoma Study. NHS, Natural History Study.

## Data Availability

All data for the present study are openly available from the original publications and in the supplementary table.

## Supplementary Materials

The following supporting information can be downloaded at: https://www.mdpi.com/article/10.3390/cancers16223839/s1; Figure S1: Illustration of data collection from the original curves. Table S1: Reverse-Engineered Raw data for the COMS and NHS cohorts. Reference [1] is cited in the supplementary materials.

## References

[1] Straatsma B.R., Diener-West M., Caldwell R., Engstrom R.E., Collaborative Ocular Melanoma Study G. (2003). Mortality after deferral of treatment or no treatment for choroidal melanoma. Am. J. Ophthalmol..

[2] Diener-West M., Earle J.D., Fine S.L., Hawkins B.S., Moy C.S., Reynolds S.M., Schachat A.P., Straatsma B.R., Collaborative Ocular Melanoma Study Group (2001). The COMS randomized trial of iodine 125 brachytherapy for choroidal melanoma, III: Initial mortality findings. COMS Report No. 18. Arch. Ophthalmol..

[3] Damato B. (2010). Does ocular treatment of uveal melanoma influence survival?. Br. J. Cancer.

[4] Damato B., Coupland S. (2009). Does delayed treatment shorten the life of patients with fatal choroidal melanoma?. Acta Ophthalmol..

[5] Stalhammar G. (2024). Delays between Uveal Melanoma Diagnosis and Treatment Increase the Risk of Metastatic Death. Ophthalmology.

[6] Hussain R., Coupland S.E., Heimann H., Eleuteri A. (2024). Re: Stalhammar G: Delays between uveal melanoma diagnosis and treatment increase the risk of metastatic death. Ophthalmology.

[7] Harbour J.W., Correa Z.M., Stacey A.W. (2024). Do Short Delays in Treatment Affect Uveal Melanoma Prognosis?. Ophthalmology.

[8] Stalhammar G., Hagstrom A., Conradi M.E., Williams P.A. Choroidal nevi and melanoma doubling times and implications for delays in treatment: A systematic review and meta-analysis. Surv. Ophthalmol..

[9] The Collaborative Ocular Melanoma Study Group (1993). Design and methods of a clinical trial for a rare condition: The Collaborative Ocular Melanoma Study. COMS Report No. 3. Control. Clin. Trials.

[10] Collaborative Ocular Melanoma Study G. (2003). Comparison of clinical, echographic, and histopathological measurements from eyes with medium-sized choroidal melanoma in the collaborative ocular melanoma study: COMS report no. 21. Arch. Ophthalmol..

[11] Diener-West M., Earle J.D., Fine S.L., Hawkins B.S., Moy C.S., Reynolds S.M., Schachat A.P., Straatsma B.R., Collaborative Ocular Melanoma Study Group (2001). The COMS randomized trial of iodine 125 brachytherapy for choroidal melanoma, II: Characteristics of patients enrolled and not enrolled. COMS Report No. 17. Arch. Ophthalmol..

[12] COMS (2006). The COMS Randomized Trial of Iodine 125 Brachytherapy for Choroidal Melanoma: V. Twelve-Year Mortality Rates and Prognostic Factors: COMS Report No. 28. Arch. Ophthalmol..

[13] Earle C.C., Pham B., Wells G.A. (2000). An assessment of methods to combine published survival curves. Med. Decis. Mak..

[14] The Swedish Riksdag (2003). The Ethical Review Act 2003:460.

[15] Singh A.D., Zabor E.C., Radivoyevitch T. (2021). Estimating Cured Fractions of Uveal Melanoma. JAMA Ophthalmol..

[16] Stalhammar G. (2023). Comprehensive causes of death in uveal melanoma: Mortality in 1530 consecutively diagnosed patients followed until death. JNCI Cancer Spectr..

[17] Khoja L., Atenafu E.G., Suciu S., Leyvraz S., Sato T., Marshall E., Keilholz U., Zimmer L., Patel S.P., Piperno-Neumann S. (2019). Meta-analysis in metastatic uveal melanoma to determine progression free and overall survival benchmarks: An international rare cancers initiative (IRCI) ocular melanoma study. Ann. Oncol..

[18] Shields C.L., Dalvin L.A., Ancona-Lezama D., Yu M.D., Di Nicola M., Williams B.K., Lucio-Alvarez J.A., Ang S.M., Maloney S., Welch R.J. (2019). Choroidal nevus imaging features in 3 806 cases and risk factors for transformation into melanoma in 2355 cases: The 2020 Taylor R. Smith and Victor T. Curtin Lecture. Retina.

[19] Dalvin L.A., Shields C.L., Ancona-Lezama D.A., Yu M.D., Di Nicola M., Williams B.K., Lucio-Alvarez J.A., Ang S.M., Maloney S.M., Welch R.J. (2019). Combination of multimodal imaging features predictive of choroidal nevus transformation into melanoma. Br. J. Ophthalmol..

[20] Sabazade S., Lumia Michalski M., Bartoszek J., Fili M., Holmström M., Stålhammar G. (2025). Development and Validation of a Deep Learning Algorithm for Differentiation of Choroidal Nevi from Small Melanoma in Fundus Photographs. Ophthalmol. Sci..

[21] Hagström A., Witzenhausen H., Stålhammar G. Tailoring Surveillance Imaging in Uveal Melanoma Based on Individual Metastatic Risk. Can. J. Ophthalmol..

[22] Decatur C.L., Ong E., Garg N., Anbunathan H., Bowcock A.M., Field M.G., Harbour J.W. (2016). Driver Mutations in Uveal Melanoma: Associations With Gene Expression Profile and Patient Outcomes. JAMA Ophthalmol..

[23] Grisanti S., Schindler F., Merz H., Kakkassery V., Sonntag S.R., Tura A. (2023). Detection of Circulating Tumor Cells in Patients with Small Choroidal Melanocytic Lesions. Ophthalmology.

[24] Augsburger J.J., Vrabec T.R. (1993). Impact of delayed treatment in growing posterior uveal melanomas. Arch. Ophthalmol..

[25] Field M.G., Durante M.A., Anbunathan H., Cai L.Z., Decatur C.L., Bowcock A.M., Kurtenbach S., Harbour J.W. (2018). Punctuated evolution of canonical genomic aberrations in uveal melanoma. Nat. Commun..

[26] Singh A.D. (2001). Uveal melanoma: Implications of tumor doubling time. Ophthalmology.

[27] Eskelin S., Pyrhönen S., Summanen P., Hahka-Kemppinen M., Kivelä T. (2000). Tumor doubling times in metastatic malignant melanoma of the uvea: Tumor progression before and after treatment. Ophthalmology.

[28] Shain A.H., Bagger M.M., Yu R., Chang D., Liu S., Vemula S., Weier J.F., Wadt K., Heegaard S., Bastian B.C. (2019). The genetic evolution of metastatic uveal melanoma. Nat. Genet..

[29] Kujala E., Mäkitie T., Kivelä T. (2003). Very long-term prognosis of patients with malignant uveal melanoma. Investig. Ophthalmol. Vis. Sci..

[30] Shields C.L., Furuta M., Thangappan A., Nagori S., Mashayekhi A., Lally D.R., Kelly C.C., Rudich D.S., Nagori A., Wakade O.A. (2009). Metastasis of uveal melanoma millimeter-by-millimeter in 8033 consecutive eyes. Arch. Ophthalmol..

