# Supplementary material for: Survival Benefit of Primary Tumor Treatment in Uveal Melanoma: A Re-Analysis of the Collaborative Ocular Melanoma Study (COMS) and Natural History Study (NHS) Cohorts
Source: Cancers (Basel). 2024 Nov 15;16(22):3839. doi: 10.3390/cancers16223839 (PMC11593181; doi:10.3390/cancers16223839)
Supplement: Supplementary file 1 [file cancers-16-03839-s001.zip › cancers-3269255-supplementary.pdf]

Supplementary Materials

Survival Benefit of Primary Tumor Treatment in Uveal Melanoma: A Re-analysis of COMS and NHS Cohorts

Hans Witzenhausen[1], Gustav Stålhammar[1,2,3]

[1]St. Erik Ophthalmic Pathology Laboratory, St. Erik Eye Hospital, Stockholm, Sweden
[2]Department of Clinical Neuroscience, Division of Eye and Vision, Karolinska Institutet, Stockholm, Sweden
[3]Ocular Oncology Service, St. Erik Eye Hospital, Stockholm, Sweden

Contents:

Supplemental Figure S1. Illustration of data collection from the original curves 2

Supplemental Table S1. Reverse-Engineered Raw data for the COMS and NHS cohorts 3

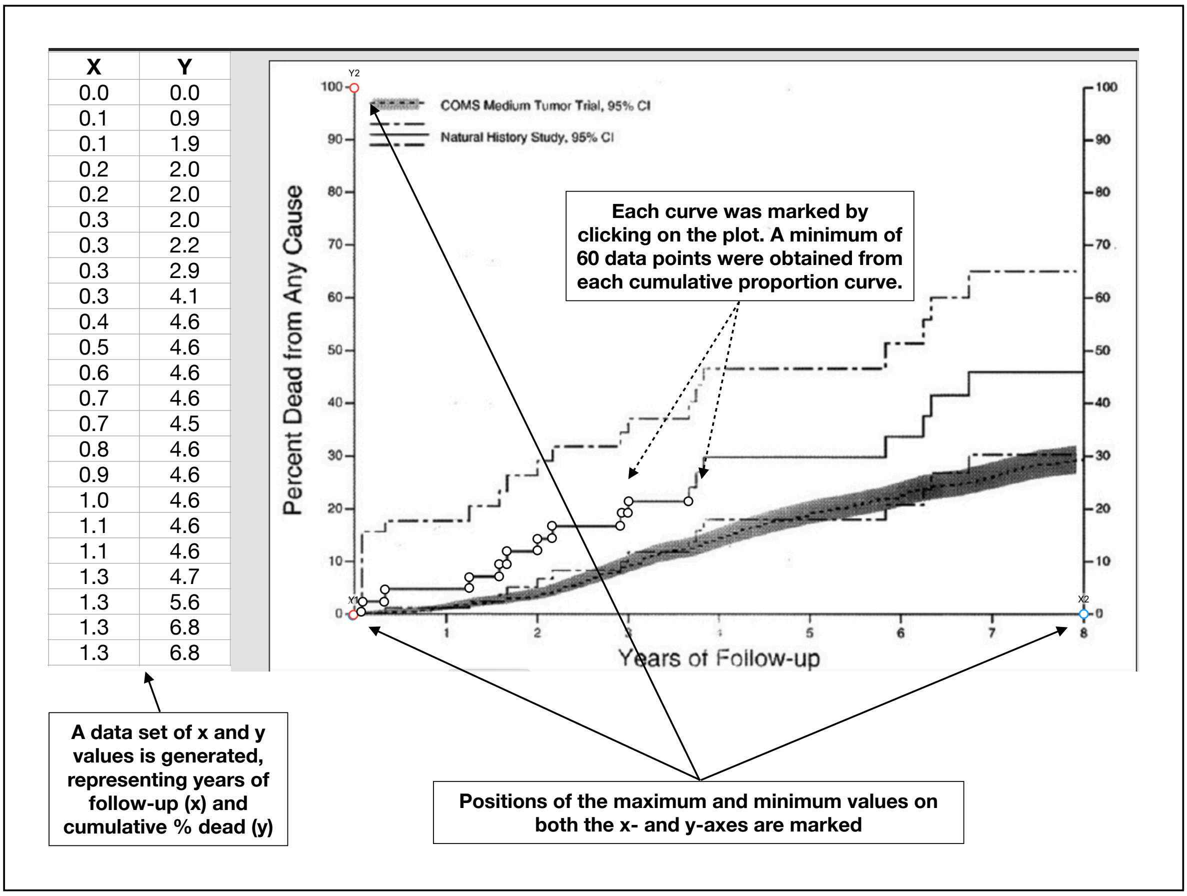

X	Y
0.0	0.0
0.1	0.9
0.1	1.9
0.2	2.0
0.2	2.0
0.3	2.0
0.3	2.2
0.3	2.9
0.3	4.1
0.4	4.6
0.5	4.6
0.6	4.6
0.7	4.6
0.7	4.5
0.8	4.6
0.9	4.6
1.0	4.6
1.1	4.6
1.1	4.6
1.3	4.7
1.3	5.6
1.3	6.8
1.3	6.8

Supplemental Figure S1.

Illustration of data collection from the original curves. Figure 2 from the original publication (titled "Kaplan-Meier curves of the cumulative proportion of patients with medium choroidal melanoma who died over time after determination of eligibility for the COMS medium melanoma trial; enrolled COMS medium tumor trial patients and NHS patients with 95% CI," reprinted from *American Journal of Ophthalmology*, volume 136, Straatsma et al., "Mortality after deferral of treatment or no treatment for choroidal melanoma," page 52, 2003, with permission from Elsevier) was uploaded to the Plot Digitizer Online App (version 3.1.5, https://plotdigitizer.com/app, accessed on September 6, 2024). This tool facilitated the extraction of data from the graphs. A minimum of 60 data points were obtained from each cumulative proportion curve for both the NHS and COMS cohorts. Two datasets were generated, each containing x-values (years after enrollment or decline of enrollment) and y-values (cumulative percentage of deaths from any cause), resulting in a dataset representing 1317 COMS patients and 42 NHS patients, with mortality proportions identical to the original figure.

Supplemental Table S1. Reverse-Engineered Raw data for the COMS and NHS cohorts.

Time	Death 0 no 1 yes	Cohort 0 COMS 1 NHS
0.5	1	0
0.5	1	0
0.5	1	0
0.5	1	0
0.7	1	0
0.7	1	0
0.7	1	0
0.8	1	0
0.8	1	0
0.8	1	0
0.8	1	0
1	1	0
1	1	0
1	1	0
1.1	1	0
1.1	1	0
1.1	1	0
1.1	1	0
1.3	1	0
1.3	1	0
1.3	1	0
1.3	1	0
1.3	1	0
1.3	1	0
1.3	1	0
1.3	1	0
1.3	1	0
1.3	1	0
1.3	1	0
1.3	1	0
1.4	1	0
1.4	1	0
1.6	1	0
1.6	1	0
1.6	1	0
1.6	1	0
1.6	1	0
1.7	1	0
1.8	1	0
1.8	1	0
1.8	1	0
2	1	0
2	1	0
2	1	0
2	1	0
2	1	0
2	1	0
2	1	0
2	1	0
2.2	1	0
2.2	1	0
2.2	1	0
2.2	1	0
2.3	1	0
2.3	1	0
2.3	1	0
2.3	1	0
2.3	1	0
2.3	1	0
2.3	1	0
2.3	1	0
2.3	1	0
2.3	1	0
2.4	1	0
2.4	1	0
2.4	1	0
2.4	1	0
2.4	1	0
2.4	1	0
2.4	1	0
2.4	1	0
2.5	1	0

2.5	1	0
2.5	1	0
2.5	1	0
2.6	1	0
2.6	1	0
2.6	1	0
2.6	1	0
2.6	1	0
2.6	1	0
2.6	1	0
2.7	1	0
2.7	1	0
2.7	1	0
2.7	1	0
2.7	1	0
2.7	1	0
2.7	1	0
2.7	1	0
2.7	1	0
2.7	1	0
2.7	1	0
2.7	1	0
2.7	1	0
2.7	1	0
2.7	1	0
2.7	1	0
2.7	1	0
2.9	1	0
2.9	1	0
2.9	1	0
2.9	1	0
2.9	1	0
2.9	1	0
2.9	1	0
2.9	1	0
2.9	1	0
3	1	0
3	1	0
3	1	0
3	1	0
3	1	0
3	1	0
3	1	0
3	1	0
3	1	0
3.1	1	0
3.1	1	0
3.1	1	0
3.1	1	0
3.1	1	0
3.2	1	0
3.2	1	0
3.2	1	0
3.2	1	0
3.2	1	0
3.2	1	0
3.2	1	0
3.2	1	0
3.2	1	0
3.2	1	0
3.2	1	0
3.2	1	0
3.2	1	0
3.2	1	0
3.3	1	0
3.3	1	0
3.3	1	0
3.3	1	0
3.3	1	0
3.4	1	0
3.4	1	0
3.4	1	0
3.4	1	0
3.4	1	0
3.4	1	0
3.4	1	0
3.4	1	0

3.4	1	0
3.5	1	0
3.5	1	0
3.5	1	0
3.5	1	0
3.7	1	0
3.7	1	0
3.7	1	0
3.7	1	0
3.7	1	0
3.8	1	0
3.8	1	0
3.8	1	0
3.8	1	0
3.8	1	0
3.8	1	0
3.8	1	0
3.8	1	0
3.8	1	0
3.8	1	0
4	1	0
4	1	0
4	1	0
4	1	0
4	1	0
4	1	0
4	1	0
4	1	0
4	1	0
4	1	0
4	1	0
4	1	0
4	1	0
4	1	0
4	1	0
4	1	0
4	1	0
4	1	0
4	1	0
4	1	0
4	1	0
4.1	1	0
4.1	1	0
4.1	1	0
4.1	1	0
4.1	1	0
4.1	1	0
4.2	1	0
4.2	1	0
4.2	1	0
4.2	1	0
4.2	1	0
4.2	1	0
4.2	1	0
4.3	1	0
4.3	1	0
4.3	1	0
4.3	1	0
4.3	1	0
4.4	1	0
4.4	1	0
4.4	1	0
4.4	1	0
4.4	1	0
4.4	1	0
4.4	1	0
4.5	1	0
4.5	1	0
4.5	1	0
4.5	1	0
4.5	1	0
4.5	1	0
4.5	1	0
4.5	1	0
4.5	1	0
4.5	1	0
4.7	1	0

4.7	1	0
4.7	1	0
4.7	1	0
4.7	1	0
4.7	1	0
4.7	1	0
4.7	1	0
4.8	1	0
4.8	1	0
4.8	1	0
4.8	1	0
4.9	1	0
4.9	1	0
4.9	1	0
4.9	1	0
4.9	1	0
5	1	0
5	1	0
5	1	0
5	1	0
5	1	0
5	1	0
5.1	1	0
5.1	1	0
5.1	1	0
5.1	1	0
5.1	1	0
5.1	1	0
5.1	1	0
5.2	1	0
5.2	1	0
5.3	1	0
5.3	1	0
5.3	1	0
5.3	1	0
5.3	1	0
5.4	1	0
5.4	1	0
5.4	1	0
5.4	1	0
5.5	1	0
5.5	1	0
5.5	1	0
5.5	1	0
5.5	1	0
5.5	1	0
5.5	1	0
5.5	1	0
5.7	1	0
5.7	1	0
5.7	1	0
5.7	1	0
5.7	1	0
5.7	1	0
5.7	1	0
5.7	1	0
5.7	1	0
5.7	1	0
5.7	1	0
5.7	1	0
5.9	1	0
5.9	1	0
6	1	0
6	1	0
6	1	0
6	1	0
6	1	0
6	1	0
6	1	0
6	1	0
6	1	0
6	1	0
6	1	0
6	1	0
6.2	1	0
6.2	1	0
6.2	1	0

6.2	1	0
6.2	1	0
6.2	1	0
6.2	1	0
6.2	1	0
6.2	1	0
6.3	1	0
6.3	1	0
6.4	1	0
6.4	1	0
6.4	1	0
6.4	1	0
6.4	1	0
6.4	1	0
6.5	1	0
6.5	1	0
6.5	1	0
6.5	1	0
6.5	1	0
6.5	1	0
6.7	1	0
6.8	1	0
6.8	1	0
6.8	1	0
6.8	1	0
6.8	1	0
6.8	1	0
6.8	1	0
6.8	1	0
6.8	1	0
6.8	1	0
6.8	1	0
6.8	1	0
7	1	0
7	1	0
7	1	0
7	1	0
7	1	0
7.1	1	0
7.1	1	0
7.1	1	0
7.1	1	0
7.1	1	0
7.1	1	0
7.1	1	0
7.2	1	0
7.2	1	0
7.2	1	0
7.2	1	0
7.2	1	0
7.2	1	0
7.3	1	0
7.3	1	0
7.3	1	0
7.3	1	0
7.3	1	0
7.3	1	0
7.4	1	0
7.4	1	0
7.4	1	0
7.4	1	0
7.4	1	0
7.4	1	0
7.5	1	0
7.5	1	0
7.5	1	0
7.5	1	0
7.5	1	0
7.5	1	0
7.5	1	0
7.7	1	0
7.7	1	0
7.7	1	0
7.7	1	0
7.8	1	0
8	1	0
8	1	0

8	1	0
8	1	0
1	0	0
1	0	0
1	0	0
1	0	0
1	0	0
1	0	0
1	0	0
1	0	0
1	0	0
3	0	0
3	0	0
3	0	0
3	0	0
3	0	0
3	0	0
3	0	0
3	0	0
3	0	0
3	0	0
3	0	0
3	0	0
5	0	0
5	0	0
5	0	0
5	0	0
5	0	0
5	0	0
5	0	0
5	0	0
5	0	0
5	0	0
5	0	0
5	0	0
5	0	0
5	0	0
5	0	0
5	0	0
5	0	0
5	0	0
5	0	0
5	0	0
5	0	0
5	0	0
5	0	0
5	0	0
5	0	0
5	0	0
5	0	0
5	0	0
5	0	0
5	0	0
5	0	0
5	0	0
5	0	0
5	0	0
5	0	0
5	0	0
5	0	0
5	0	0
5	0	0
5	0	0
5	0	0
5	0	0
5	0	0
5	0	0
5	0	0
5	0	0
5	0	0
5	0	0
5	0	0
5	0	0
5	0	0
6	0	0
6	0	0
6	0	0

6	0	0
6	0	0
6	0	0
6	0	0
6	0	0
6	0	0
6	0	0
6	0	0
6	0	0
6	0	0
6	0	0
6	0	0
6	0	0
6	0	0
6	0	0
6	0	0
6	0	0
6	0	0
6	0	0
6	0	0
6	0	0
6	0	0
6	0	0
6	0	0
6	0	0
6	0	0
6	0	0
6	0	0
6	0	0
6	0	0
6	0	0
6	0	0
6	0	0
6	0	0
6	0	0
6	0	0
6	0	0
6	0	0
6	0	0
6	0	0
6	0	0
6	0	0
6	0	0
6	0	0
6	0	0
6	0	0
6	0	0
6	0	0
6	0	0
6	0	0
6	0	0
6	0	0
6	0	0
6	0	0
6	0	0
6	0	0
6	0	0
6	0	0
6	0	0
6	0	0
6	0	0
6	0	0
6	0	0
6	0	0
6	0	0
6	0	0
6	0	0
6	0	0
6	0	0
6	0	0
6	0	0
6	0	0
6	0	0
6	0	0
6	0	0
6	0	0
6	0	0

6	0	0
6	0	0
6	0	0
6	0	0
6	0	0
6	0	0
6	0	0
6	0	0
6	0	0
6	0	0
6	0	0
6	0	0
6	0	0
6	0	0
6	0	0
6	0	0
6	0	0
6	0	0
6	0	0
6	0	0
6	0	0
6	0	0
6	0	0
6	0	0
6	0	0
6	0	0
6	0	0
6	0	0
6	0	0
6	0	0
6	0	0
6	0	0
6	0	0
6	0	0
6	0	0
6	0	0
6	0	0
6	0	0
6	0	0
6	0	0
6	0	0
6	0	0
6	0	0
6	0	0
6	0	0
6	0	0
6	0	0
6	0	0
6	0	0
6	0	0
6	0	0
6	0	0
6	0	0
6	0	0
6	0	0
6	0	0
6	0	0
6	0	0
6	0	0
6	0	0
6	0	0
6	0	0
6	0	0
6	0	0
6	0	0
6	0	0
11	0	0
11	0	0
11	0	0
11	0	0
11	0	0
11	0	0
11	0	0
11	0	0
11	0	0
11	0	0
11	0	0

11	0	0
11	0	0
11	0	0
11	0	0
11	0	0
11	0	0
11	0	0
11	0	0
11	0	0
11	0	0
11	0	0
11	0	0
11	0	0
11	0	0
11	0	0
11	0	0
11	0	0
11	0	0
11	0	0
11	0	0
11	0	0
11	0	0
11	0	0
11	0	0
11	0	0
11	0	0
11	0	0
11	0	0
11	0	0
11	0	0
11	0	0
11	0	0
11	0	0
11	0	0
11	0	0
11	0	0
11	0	0
11	0	0
11	0	0
11	0	0
11	0	0
11	0	0
11	0	0
11	0	0
11	0	0
11	0	0
11	0	0
11	0	0
11	0	0
11	0	0
11	0	0
11	0	0
11	0	0
11	0	0
11	0	0
11	0	0
11	0	0
11	0	0
11	0	0
11	0	0
11	0	0
11	0	0
11	0	0
11	0	0
11	0	0
11	0	0
11	0	0
11	0	0
11	0	0
11	0	0
11	0	0
11	0	0
11	0	0
11	0	0
11	0	0
11	0	0
11	0	0

11	0	0
11	0	0
11	0	0
11	0	0
11	0	0
11	0	0
11	0	0
11	0	0
11	0	0
11	0	0
11	0	0
11	0	0
11	0	0
11	0	0
11	0	0
11	0	0
11	0	0
11	0	0
11	0	0
11	0	0
11	0	0
11	0	0
11	0	0
11	0	0
11	0	0
11	0	0
11	0	0
11	0	0
11	0	0
11	0	0
11	0	0
11	0	0
11	0	0
11	0	0
11	0	0
11	0	0
11	0	0
11	0	0
11	0	0
11	0	0
11	0	0
11	0	0
11	0	0
11	0	0
11	0	0
11	0	0
11	0	0
11	0	0
11	0	0
11	0	0
11	0	0
11	0	0
11	0	0
11	0	0
11	0	0
11	0	0
11	0	0
11	0	0
11	0	0
11	0	0
11	0	0
11	0	0
11	0	0
11	0	0
11	0	0
11	0	0
11	0	0
11	0	0
11	0	0
11	0	0
11	0	0
11	0	0
11	0	0
11	0	0
11	0	0
11	0	0
11	0	0

11	0	0
11	0	0
11	0	0
11	0	0
11	0	0
11	0	0
11	0	0
11	0	0
11	0	0
11	0	0
11	0	0
11	0	0
11	0	0
11	0	0
11	0	0
11	0	0
11	0	0
11	0	0
11	0	0
11	0	0
11	0	0
11	0	0
11	0	0
11	0	0
11	0	0
11	0	0
11	0	0
11	0	0
11	0	0
11	0	0
11	0	0
11	0	0
11	0	0
11	0	0
11	0	0
11	0	0
11	0	0
11	0	0
11	0	0
11	0	0
11	0	0
11	0	0
11	0	0
11	0	0
11	0	0
11	0	0
11	0	0
11	0	0
11	0	0
11	0	0
11	0	0
11	0	0
11	0	0
11	0	0
11	0	0
11	0	0
11	0	0
11	0	0
11	0	0
11	0	0
11	0	0
11	0	0
11	0	0
11	0	0
11	0	0
11	0	0
11	0	0
11	0	0
11	0	0
11	0	0
11	0	0
11	0	0
11	0	0
11	0	0
11	0	0
11	0	0
11	0	0

11	0	0
11	0	0
11	0	0
11	0	0
11	0	0
11	0	0
11	0	0
11	0	0
11	0	0
11	0	0
11	0	0
11	0	0
11	0	0
11	0	0
11	0	0
11	0	0
11	0	0
11	0	0
11	0	0
11	0	0
11	0	0
11	0	0
11	0	0
11	0	0
11	0	0
11	0	0
11	0	0
11	0	0
11	0	0
11	0	0
11	0	0
11	0	0
11	0	0
11	0	0
11	0	0
11	0	0
11	0	0
11	0	0
11	0	0
11	0	0
11	0	0
11	0	0
11	0	0
11	0	0
11	0	0
11	0	0
11	0	0
11	0	0
11	0	0
11	0	0
11	0	0
11	0	0
11	0	0
11	0	0
11	0	0
11	0	0
11	0	0
11	0	0
11	0	0
11	0	0
11	0	0
11	0	0
11	0	0
11	0	0
11	0	0
11	0	0
11	0	0
11	0	0
11	0	0
11	0	0
11	0	0
11	0	0
11	0	0
11	0	0
11	0	0
11	0	0
11	0	0

11	0	0
11	0	0
11	0	0
11	0	0
11	0	0
11	0	0
11	0	0
11	0	0
11	0	0
11	0	0
11	0	0
11	0	0
11	0	0
11	0	0
11	0	0
11	0	0
11	0	0
11	0	0
11	0	0
11	0	0
11	0	0
11	0	0
11	0	0
11	0	0
11	0	0
11	0	0
11	0	0
11	0	0
11	0	0
11	0	0
11	0	0
11	0	0
11	0	0
11	0	0
11	0	0
11	0	0
11	0	0
11	0	0
11	0	0
11	0	0
11	0	0
11	0	0
11	0	0
11	0	0
11	0	0
11	0	0
11	0	0
11	0	0
11	0	0
11	0	0
11	0	0
11	0	0
11	0	0
11	0	0
11	0	0
11	0	0
11	0	0
11	0	0
11	0	0
11	0	0
11	0	0
11	0	0
11	0	0
11	0	0
11	0	0
11	0	0
11	0	0
11	0	0
11	0	0
11	0	0
11	0	0
11	0	0
11	0	0
11	0	0
11	0	0
11	0	0
11	0	0

11	0	0
11	0	0
11	0	0
11	0	0
11	0	0
11	0	0
11	0	0
11	0	0
11	0	0
11	0	0
11	0	0
11	0	0
11	0	0
11	0	0
11	0	0
11	0	0
11	0	0
11	0	0
11	0	0
11	0	0
11	0	0
11	0	0
11	0	0
11	0	0
11	0	0
11	0	0
11	0	0
11	0	0
11	0	0
11	0	0
11	0	0
11	0	0
11	0	0
11	0	0
11	0	0
11	0	0
11	0	0
11	0	0
11	0	0
11	0	0
11	0	0
11	0	0
11	0	0
11	0	0
11	0	0
11	0	0
11	0	0
11	0	0
11	0	0
11	0	0
11	0	0
11	0	0
11	0	0
11	0	0
11	0	0
11	0	0
11	0	0
11	0	0
11	0	0
11	0	0
11	0	0
11	0	0
11	0	0
11	0	0
11	0	0
11	0	0
11	0	0
11	0	0
11	0	0
11	0	0
11	0	0
11	0	0
11	0	0
11	0	0
11	0	0
11	0	0
11	0	0

11	0	0
11	0	0
11	0	0
11	0	0
11	0	0
11	0	0
11	0	0
11	0	0
11	0	0
11	0	0
11	0	0
11	0	0
11	0	0
11	0	0
11	0	0
11	0	0
11	0	0
11	0	0
11	0	0
11	0	0
11	0	0
11	0	0
11	0	0
11	0	0
11	0	0
11	0	0
11	0	0
11	0	0
11	0	0
11	0	0
11	0	0
11	0	0
11	0	0
11	0	0
11	0	0
11	0	0
11	0	0
11	0	0
11	0	0
11	0	0
11	0	0
11	0	0
11	0	0
11	0	0
11	0	0
11	0	0
11	0	0
11	0	0
11	0	0
11	0	0
11	0	0
11	0	0
11	0	0
11	0	0
11	0	0
11	0	0
11	0	0
11	0	0
11	0	0
11	0	0
11	0	0
11	0	0
11	0	0
11	0	0
11	0	0
11	0	0
11	0	0
11	0	0
11	0	0
11	0	0
11	0	0
11	0	0
11	0	0
11	0	0
11	0	0
11	0	0
11	0	0

11	0	0
11	0	0
11	0	0
11	0	0
11	0	0
11	0	0
11	0	0
11	0	0
11	0	0
11	0	0
11	0	0
11	0	0
11	0	0
11	0	0
11	0	0
11	0	0
11	0	0
11	0	0
11	0	0
11	0	0
11	0	0
11	0	0
11	0	0
11	0	0
11	0	0
11	0	0
11	0	0
11	0	0
11	0	0
11	0	0
11	0	0
11	0	0
11	0	0
11	0	0
11	0	0
11	0	0
11	0	0
11	0	0
11	0	0
11	0	0
11	0	0
11	0	0
11	0	0
11	0	0
11	0	0
11	0	0
11	0	0
11	0	0
11	0	0
11	0	0
11	0	0
11	0	0
11	0	0
11	0	0
11	0	0
11	0	0
11	0	0
11	0	0
11	0	0
11	0	0
11	0	0
11	0	0
11	0	0
11	0	0
11	0	0
11	0	0
11	0	0
11	0	0
11	0	0
11	0	0
11	0	0
11	0	0
11	0	0
11	0	0
11	0	0
11	0	0
11	0	0

11	0	0
11	0	0
11	0	0
11	0	0
11	0	0
11	0	0
11	0	0
11	0	0
11	0	0
11	0	0
11	0	0
11	0	0
11	0	0
11	0	0
11	0	0
11	0	0
11	0	0
11	0	0
11	0	0
11	0	0
11	0	0
11	0	0
11	0	0
11	0	0
11	0	0
11	0	0
11	0	0
11	0	0
11	0	0
11	0	0
11	0	0
11	0	0
11	0	0
11	0	0
11	0	0
11	0	0
11	0	0
11	0	0
11	0	0
11	0	0
11	0	0
11	0	0
11	0	0
11	0	0
11	0	0
11	0	0
11	0	0
11	0	0
11	0	0
11	0	0
11	0	0
11	0	0
11	0	0
11	0	0
11	0	0
11	0	0
11	0	0
11	0	0
11	0	0
11	0	0
11	0	0
11	0	0
11	0	0
11	0	0
11	0	0
11	0	0
11	0	0
11	0	0
11	0	0
11	0	0
11	0	0
11	0	0
11	0	0
11	0	0
11	0	0
11	0	0
11	0	0

11	0	0
11	0	0
11	0	0
11	0	0
11	0	0
11	0	0
11	0	0
11	0	0
11	0	0
11	0	0
11	0	0
11	0	0
11	0	0
0.1	1	1
0.3	1	1
1.3	1	1
1.6	1	1
1.7	1	1
2	1	1
2.2	1	1
2.9	1	1
3	1	1
3.6	1	1
3.7	1	1
3.8	1	1
11	0	1
4	1	1
5.8	1	1
6.3	1	1
6.8	1	1
6.7	0	1
4	0	1
4.11	0	1
4.22	0	1
4.33	0	1
4.44	0	1
4.56	0	1
4.67	0	1
4.78	0	1
4.89	0	1
5	0	1
5.2	0	1
5.4	0	1
5.6	0	1
5.8	0	1
6	0	1
6.3	0	1
6.6	0	1
7	0	1
7.5	0	1
8	0	1
9	0	1
10.7	0	1
11	0	1
11	0	1
